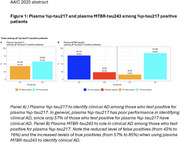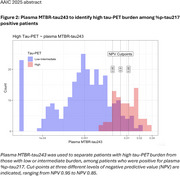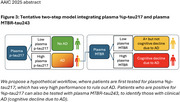# Clinical usefulness of plasma *p*‐tau217 and eMTBR‐tau243 combination for diagnosis and patient stratification

**DOI:** 10.1002/alz70856_100822

**Published:** 2025-12-25

**Authors:** Niklas Mattsson‐Carlgren, Sebastian Palmqvist, Kanta Horie, Nicolas R. Barthélemy, Gemma Salvadó, Shorena Janelidze, Lyduine E. Collij, Suzanne E. Schindler, Erik Stomrud, Randall J. Bateman, Oskar Hansson

**Affiliations:** ^1^ Clinical Memory Research Unit, Department of Clinical Sciences Malmö, Faculty of Medicine, Lund University, Sweden, Lund, Sweden; ^2^ Clinical Memory Research Unit, Department of Clinical Sciences Malmö, Faculty of Medicine, Lund University, Lund, Sweden; ^3^ Washington University School of Medicine, St. Louis, MO, USA; ^4^ Department of Neurology, Washington University in St. Louis School of Medicine, St. Louis, MO, USA; ^5^ Clinical Memory Research Unit, Department of Clinical Sciences, Lund University, Lund, Sweden; ^6^ Washington University in St. Louis, St. Louis, MO, USA

## Abstract

**Background:**

AD can be identified with early biomarkers (core 1 biomarkers), e.g., plasma *p*‐tau217. Abnormalities in biomarkers that change later (core 2 biomarkers) may increase confidence that AD is contributing to cognitive symptoms. Plasma eMTBR‐tau243 is a promising blood biomarker (BBM) for tau tangle pathology (proposed core 2 biomarker). It is unclear how plasma MTBR‐tau243 can be used together with *p*‐tau217 to support diagnosis and patient stratification.

**Method:**

We included 571 patients with cognitive symptoms from the Swedish BioFINDER‐2 study (142 subjective cognitive decline, 258 mild cognitive impairment, 171 dementia patients). Plasma %p‐tau217 and eMTBR‐tau243 were examined. The primary outcome was clinical AD (confirmed with Ab‐positivity and tau‐PET‐positivity). Secondary, we evaluated plasma MTBR‐tau243 to identify high neocortical tau‐PET burden among %p‐tau217 positive patients.

**Result:**

35% (*N* = 201) had clinical AD, 61% (*N* = 349) were positive for %p‐tau217, and 34% (*N* = 196) were positive for eMTBR‐tau243. Clinical AD was very rare among %p‐tau217 negative patients.

Among %p‐tau217 positive patients, 99% (*N* = 346) were Ab positive and 57% (*N* = 200) had clinical AD. The latter are considered true positives, while the remaining %p‐tau217 positives (43%, *N* = 149) are considered false positives for clinical AD (Figure 1A).

About half of the %p‐tau217 positives (56%, *N* = 194) were also positive for eMTBR‐tau243. Among %p‐tau217 positives, eMTBR‐tau243 had accuracy 81% to predict clinical AD, and was associated with 85% (*N* = 164) true positives, 16% (*N* = 30) false positives, 77% (*N* = 119) true negatives, and 23% (*N* = 36) false negatives (Figure 1B).

Second, we studied if eMTBR‐tau243 could identify, among %p‐tau217 positives, those with high tau‐PET load (23% of all %p‐tau217 positives), since these individuals may have less benefit from Ab‐targeting therapies. A cutoff set at NPV 90% had 76% PPV and 87% accuracy in classifying individuals with high tau‐PET load (Figure 2).

**Conclusion:**

Plasma %p‐tau217 can rule out Ab‐positivity, while eMTBR‐tau243 can rule in clinical AD. A two‐step model is proposed, with initial testing of %p‐tau217 followed by eMTBR‐tau243 in %p‐tau217‐positive patients (Figure 3). This aids in assessing whether AD pathology is either likely to be asymptomatic or is likely to be causing cognitive impairment. MTBR‐tau243 may also aid in identifying particularly high tangle burden.